# The postural stability of children with foetal alcohol spectrum disorders during one-leg stance: A feasibility study

**DOI:** 10.4102/ajod.v7i0.319

**Published:** 2018-03-29

**Authors:** Yolandi Brink, John Cockcroft, Soraya Seedat, Philip May, Wendy Kalberg, Quinette Louw

**Affiliations:** 1 Division of Physiotherapy, Stellenbosch University, South Africa; 2 Department of Psychiatry, Stellenbosch University, South Africa; 3 Department of Nutrition, University of North Carolina, United States; 4 Nutrition Research Institute, Kannapolis, United States; 5 Center on Alcoholism, Substance Abuse, and Addictions, The University of New Mexico, Mexico

## Abstract

**Background:**

Postural control may be impaired in children with foetal alcohol spectrum disorders (FASD). The study assessed the protocol feasibility in terms of (1) recruiting children with FASD in a rural, small town; (2) using the measurement instruments in a real-life setting; (3) the one-leg standing (OLS) task and (4) presenting preliminary results on postural stability of children with and without FASD.

**Methods:**

Nine-year-old children diagnosed with and without FASD were invited to participate. Twenty-eight children performed OLS. Feasibility outcomes included recruitment, measurement instrument use and task instruction. Postural stability outcomes included standing duration, centre of pressure (COP) and body segment acceleration.

**Results:**

Participants recruitment was feasible in terms of the (1) ability to sample a reasonable participant number in a rural town setting and the capacity to increase the sample size if more schools are included in the sampling frame and (2) use of assent and consent forms that were appropriate for this population. The measurement instruments were user-friendly, cost-effective and time-efficient. Instructions for the task require amendment to address foot placement of the non-weight–bearing leg. There was a significant difference between cases and controls on mean COP velocity (*p* = 0.001) and the pelvis segment acceleration in the mediolateral direction (*p* = 0.01) and the anteroposterior direction (*p* = 0.027). The control children took longer to achieve postural control. The girls demonstrated a significant difference for the COP anteroposterior displacement (*p* = 0.008) and velocity (*p* = 0.049).

**Conclusions:**

The recruitment of children with and without FASD in a rural, small town and the administration of measurement instruments in a real-life, school-based setting was feasible. However, the verbal instructions for the task require revision. The male control group took longer to achieve postural control because the task was performed differently between the two groups. However, the case girls were slower to achieve postural control than control girls though performing the task similarly.

## Background

Foetal alcohol syndrome (FAS) represents the extreme end of a continuum of foetal alcohol spectrum disorders (FASD) and is the most common birth defect in South Africa, affecting more than one million South Africans (Bulletin of the World Health Organization 2011; Riley, Infante & Warren 2011). Extensive research of FASD, which includes FAS, partial FAS (PFAS) and alcohol-related neurodevelopmental disorder (ARND) has been conducted in rural or small-town settings in the Western Cape Province of South Africa, where 13.5%–20.8% children have been diagnosed with FASD (9.1%–10.0% with FAS; 7.0%–7.5% with PFAS; and 4.7% with ARND) (May et al. 2013; Olivier et al. 2013). This far exceeds the rates reported in other in-school studies internationally (May et al. 2009). The high occurrence of FAS in the Western Cape places a significant economic burden on the country as the estimated annual burden spent on the management of children with FAS is about 5% of the 2010/11 Department of Health’s budget (Crede et al. 2011). Because FAS is entirely preventable, preventive efforts would yield significant healthcare cost savings (Chersich et al. 2012). However, the occurrence of FAS in the wine region of the Western Cape has not reduced over the past decade (Crede et al. 2011). Therefore, cost-saving intervention strategies to improve the overall health of children with FAS are needed.

Foetal Alcohol Syndrome is diagnosed based on: growth retardation (below 10th percentile for either height or weight), central nervous system dysfunction, characteristic facial anomalies and confirmation of maternal alcohol exposure (Frost, Gist & Adriano 2011; May et al. 2013; Simmons et al. 2010). In children with FASD, neuroimaging studies have shown size reduction of brain structures, that is, the cerebellum, corpus callosum and basal ganglia (Roussotte et al. 2012; Spadoni et al. 2007). The cerebellum and the basal ganglia, which seem to be particularly vulnerable to prenatal alcohol exposure (PAE), are associated with motor functions such as posture, balance, coordination, motor programming and procedural learning (Domellof et al. 2011; Spadoni et al. 2007). Research has indicated that children with PAE or FASD display postural control deficits, decreased gross and fine motor coordination, increased postural sway and delayed temporal processing and atypical temporo-spatial trajectories of motor tasks (Adnams et al. 2001; Barr et al. 1990; Domellof et al. 2011; Kalberg et al. 2006; Kooistra et al. 2009; Roebuck et al. 1998a, 1998b; Simmons et al. 2010). These motor deficits may be related to the teratogenic effects of PAE on structures of the central nervous system. These motor deficits are likely to persist into adulthood, which underscores the importance of early detection of motor function problems related to FASD.

To our knowledge, only two studies (Kooistra et al. 2009; Roebuck et al. 1998a), incorporating the use of objective measurement (i.e. a force plate), have described postural stability of children with FASD. These researchers reported centre of pressure (COP) displacement during standing. However, the authors reported inconsistent findings. Postural stability or control refers to the ability to control the orientation of body segments and to maintain the projection of the centre of gravity (COG) within the base of support (Shumway-Cook & Woollacott 2001). This COG oscillates as a result of nonlinearities of neuromuscular control and is referred to as postural sway. COG displacements are transmitted to the support surface as a compound measure of COP (Blaszczyk, Lowe & Hansen 1994). COP parameters constitute an indirect method of describing postural control or balance and reflect the ability of the body to adjust accordingly to maintain balance. No research describing the postural stability of South African children with FASD, using objective measurement methods, has been published. Domellof et al. (2011) affirmed the importance of specialised motion analysis techniques to investigate the motor control abnormalities of FAS children. Therefore, further investigation using objective, specialised motion analytical approaches is warranted to obtain objective evidence underpinning the motor ability of children with FASD on which early screening and future interventions can be based.

The aim of the feasibility study was to assess the appropriateness and practicality of the study protocol in terms of (1) recruiting children with and without FASD in a rural, small-town setting; (2) using the measurement instruments in a real-life, school-based setting; (3) the one-leg standing (OLS) task; and (4) to present preliminary results on postural stability of children with and without FASD as a means to inform future research on sample size calculation and to describe any potential differences between the two groups.

## Methods

### Study design, setting and population

A feasibility study was conducted in a rural town in the Western Cape of South Africa. This area has a high prevalence of FASD and is an official FASD research site for a large collaborative research project. The study population consisted of 9-year-old boys and girls attending primary schools in the town or surrounding farm schools, diagnosed with FASD (cases) or with no PAE (controls). The FASD and no PAE diagnoses were based on the diagnostic procedures described by May et al. (2013), who previously screened these grade 1 learners when they were 6 years of age (May et al. 2013). The age group was conveniently chosen because, at the time of the study, most of the screened children would have reached the age of 9 years.

### Participants

#### Sampling method

We obtained the names of all 9-year-old boys and girls attending three town schools and four farm schools who were previously screened for FASD and captured on the research database of the study conducted in 2011 (May et al. 2013). The four farm schools were situated within a 10-km radius of the town. Our sampling procedure aimed for the inclusion of at least 12 children (cases) from one of the three diagnostic categories of FASD (ARND, PFAS and FAS) and 12 control children based on no PAE. An equal distribution of boys (*n* = 12) and girls (*n* = 12) was sought.

#### Inclusion and exclusion criteria

Boys and girls (1) aged 9 years; (2) diagnosed with either ARND, PFAS and FAS or no PAE when they were 6 years old; (3) still attending one of the seven selected primary schools; (4) and from whom parental or guardian and child written informed consent have been obtained were eligible to participate. Children diagnosed with neurological, musculoskeletal or movement disorders other than those associated with PAE were excluded (e.g. Attention Deficit Disorders and Developmental Coordination Disorder) (Kooistra et al. 2009).

### Measurement instruments

#### Inertial and magnetic measurement system

An inertial and magnetic measurement system (IMMS) provides three-dimensional (3D) orientation, acceleration and angular velocity data of human movement. We used a wireless IMMS (Mtw, Xsens Technologies, B.V.), which has been shown to describe human motion accurately in research and clinical settings (Guo et al. 2013; Saber-Sheikh et al. 2010; Zhou et al. 2008).

#### Dynamic pressure mapping system

Dynamic pressure mapping systems measure force distribution on a contact surface during sitting, standing or gait. We used a portable pressure-sensitive mat (Matscan, Tekscan Inc.) which is lightweight and easy to use. The Matscan software allows for real-time and offline viewing of the plantar pressure distribution and COP position when a subject is standing on the mat (Brenton-Rule et al. 2012).

### Balance task

All participants performed barefoot OLS on both legs with eyes open and closed resulting in four conditions (De Kegel et al. 2011). Each participant was given standard instructions. For the eyes-open trials, the participants were told (1) to stand on one leg for as long and as still as possible, (2) that the non-weight–bearing leg (NWB) may not touch the weight-bearing leg or touch the floor, (3) to focus on a picture approximately 3 m away at eye level, and (4) to allow the arms to hang free at the sides (De Kegel et al. 2011). If participants could not maintain a neutral hip alignment and 90° knee flexion (as demonstrated), they were allowed other one-leg stance positions as long as they adhered to the criteria mentioned above. The capture commenced when the foot lifted from the pressure mat. For the eyes-closed trials, similar instructions were given except that the participant was asked to first lift the foot and then close the eyes once steady. The capture commenced when the eyes closed (Geuze 2003).

### Feasibility outcomes

#### Recruitment

Accessibility to an adequate number of eligible participants and comprehension of assent and consent forms given to participants and parents, respectively, were defined as outcomes. We evaluated the comprehension of consent and assent forms by parents and participants based on questions that were raised during the consenting process.

#### Measurement instruments

We looked at the adaptability of the measurement instruments to different classroom set-ups and the user friendliness of the measurement instruments to the participants and the operator.

**Balance task (one-leg standing):** We considered the verbal and visual instructions given by the researcher to the participants and the actual performance of the participants observed via video recording and its correlation to the postural stability data obtained.

### Postural stability outcome measures

Table 1 lists and explains the postural stability outcome measures that were included in this study.

**TABLE 1:** The postural stability outcome measures.

Outcome measure	Definition
Time (duration)	The time variable, measured in seconds, refers to the duration of one-leg standing for each of the four conditions (left and right one-leg standing with eyes open and closed).
Centre of pressure (COP)	These four measures include the maximum COP displacement or distance travelled in the anteroposterior (AP_max_) (forwards and backwards) and the mediolateral (ML_max_) (sideways) directions and the COP mean velocity (average displacement divided by the time) in the anteroposterior (AP_vel_) and the mediolateral (ML_vel_) directions (Johnson, MacWilliams & Stevenson 2014; Kooistra et al. 2009; Zumbrunn, MacWilliams & Johnson 2011).
Accelerometry	These six measures include the root mean square (RMS) of the dynamic acceleration (rate at which the segment changes its velocity) (normalised to milligravity) of the head, thorax and pelvis segments during the trials. The RMS acceleration was decomposed in the mediolateral (ML_ACC_) (sideways) and anteroposterior (AP_ACC_) (forwards and backwards) directions, which were calibrated relative to the attached inertial and magnetic measurement system, using a standard static pose procedure (Cereatti, Trojaniello & Della Croce 2015; Morton, Baillie & Ramirez-Iniguez 2013).

### Procedure

**FIGURE 1:**
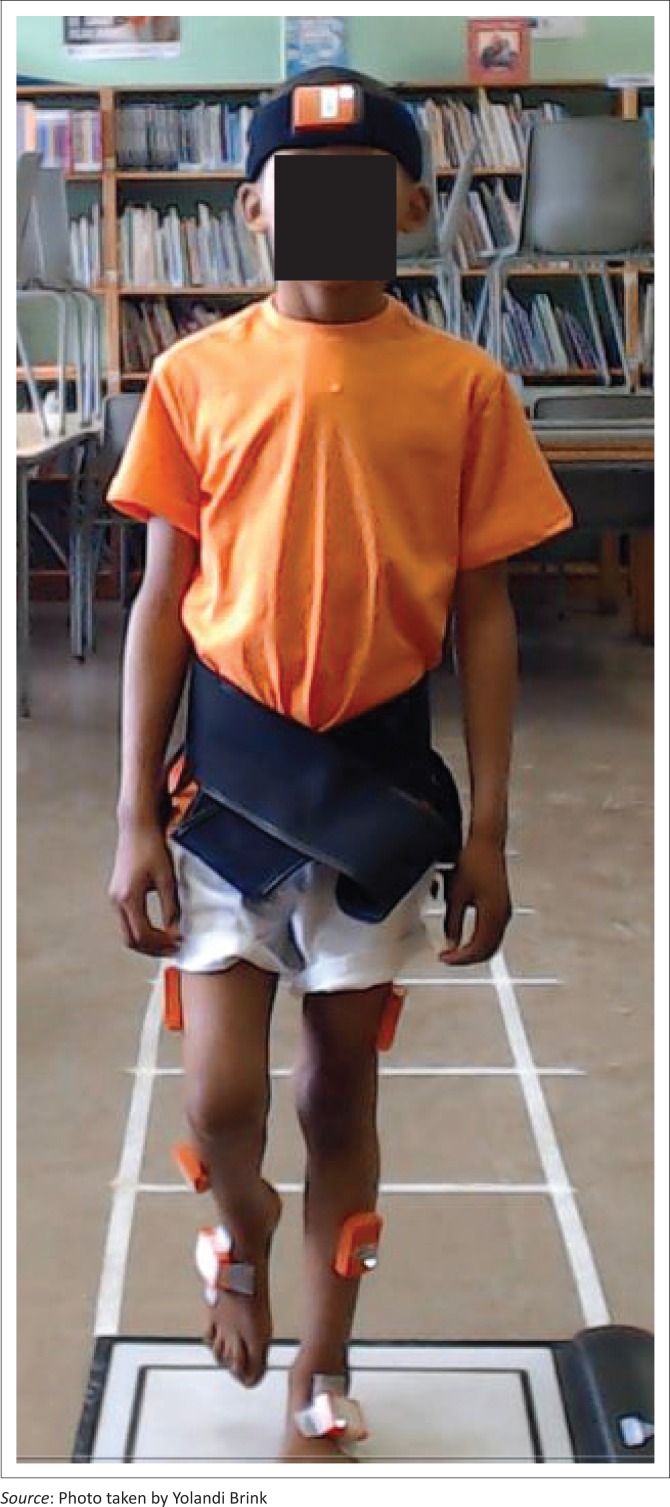
Placement of the Xsens sensors on a participant.

All eligible children were invited to participate in the study and received assent forms (for the participants) and written informed consent forms (for the parents). The postural stability measurements of those consenting participants were captured at the schools during school hours. The participants were dressed in sport shorts and T-shirts provided by the researcher. The researcher (Y.B.), blinded to the diagnosis of the participants, explained the procedure to all participants. The researchers (Y.B. and J.C.) were only informed of the children’s diagnosis once all data were captured. Height and weight measurements were taken by a research assistant. The researcher (Y.B.) attached the three inertial sensors on the head, thorax and pelvis segments using adhesive tape as shown in Figure 1. The head sensor was kept in place by a headband so that the sensor was positioned in the centre of the forehead, the thorax sensor was positioned over the manubrium using double-sided tape and the pelvic sensor was kept in position over S_1_ via a pelvic belt (Whitney et al. 2011).

The IMMS were calibrated prior to data collection to track movements of the body segments in the anatomical planes of motion. This was done using a static trial in which the participant assumed a neutral standing posture in a magnetically clean zone (Cereatti et al. 2015; Morton et al. 2013). The researcher provided verbal instructions and demonstrated the task. The participant performed the eyes-open trials first, then the eyes-closed trials and was given a choice of which leg to be tested first. The participant received two opportunities (lasting up to 20 s) for each condition (De Kegel et al. 2011; Geuze 2003).

### Data processing of postural stability outcomes

For the time variable, the duration of successful OLS was recorded observationally via the Matscan software. Time was recorded up to 20 s while the foot remained in contact with the pressure mat, and the NWB foot did not make contact with the floor.

For the COP displacement and mean velocity outcomes, the best trial was chosen based on the longest period of no shuffling (some participants changed the direction of foot placement on the mat) (Giagazoglou et al. 2013; Zumbrunn et al. 2011). If both trials were performed without any shuffling and for the full 20 s, the first trial per condition was selected for analysis. For the COP analysis, a successful eyes-open trial meant no shuffling for the first 7 s of the trial (Geuze 2003), whereas it was deemed successful for the eyes-closed trial if the participant did not shuffle for the first 3 s of the trial (Zumbrunn et al. 2011). The start and end times of the first sufficient portion of time in which no shuffling occurred were identified for each trial through visual inspection of the plantar pressure distribution. These times were exported together with the COP data to a custom Matlab algorithm, which calculated the COP range and mean velocity outcomes for the selected period of the trial data. The ML_vel_ and ML_max_ were assumed to be parallel to the axes of the Matscan system as the subjects’ feet were aligned accordingly during testing.

To obtain the accelerometry parameters, the same trials were analysed for the same duration of time as for the COP parameters. We imported the IMMS orientation data and dynamic acceleration data from the Xsens data files into a custom Matlab script. The IMMS orientation data from the static calibration trial were first used to calculate the standard sensor-to-segment coordinate frame transform. This transformation was a rotation matrix which could then be used to express the IMMS dynamic acceleration data in the respective anatomical frames of the pelvis, thorax and head. These dynamic acceleration data are already corrected for gravitational acceleration (using the Xsens IMMS sensor fusion algorithm) and were thus used directly after rotation to the segment axis to calculate the accelerometry outcomes in Matlab. There was no difference between the left and right sides for all three postural stability measures; thus, the two sides were combined for the eyes-open and -closed trials (De Kegel et al. 2011).

### Statistical analysis of postural stability outcomes

Descriptive statistics (median and ranges) were used to describe the postural stability measures (time, COP and accelerometry) for the case and control groups and per gender because the data were not normally distributed. To ascertain differences between cases and controls for the time, COP and accelerometry data, Mann–Whitney tests (for non-parametric data) were conducted, with significance level *p* < 0.05.

### Ethical considerations

Institutional ethical approval (N13/10/140) and permission from the Western Cape Department of Education were obtained. Written informed consent was obtained from the parents or guardians and the participants. The data obtained from this study were kept on a password-protected computer.

Consent to publish a photo image has been obtained from the participant and the parents.

## Results and discussion

In this study we explored the feasibility of testing postural stability in children with and without FASD using portable 3D biomechanical analysis instruments in a rural school-based setting. The results of the feasibility testing are provided and the proposed amendments to the testing protocol are described in response to the findings of the study.

### Feasibility to recruit participants

Of the 47 children diagnosed when they were 6 years old, 43 (91.5%) were attending the same schools and received written informed consent letters. All children attended mainstream schools. Thirty-one (66%) children consented to participate in the study. On the day of testing, one boy was absent and one girl and boy could not be tested within school hours and were thus excluded. Therefore, 28 children, 16 boys and 12 girls, participated in the study. In total, 6 children with FAS (4 boys; 2 girls), 4 with ARND (2 boys; 2 girls), 4 with PFAS (2 boys; 2 girls) and 14 with no PAE (8 boys; 6 girls) were assessed. We could test four boys more than what we originally planned to include in the sample. We had no difficulty in obtaining assent and consent from all participants and parents and received no questions from school principals, participants or parents on the clarity of the assent and consent forms. Participants and parents understood the content of the forms and the forms were considered appropriate for use in future larger sample studies. Thus, we do not foresee any pragmatic difficulties in recruiting subjects for a proposed larger study. Based on the preliminary findings shown in Appendix 1, we used the mean (SD) of the mediolateral (ML) velocity parameters for the group and performed a priori sample size calculation for mean difference between two independent groups. The mean (SD) for the case and control groups were 2.49 cm.s^−1^ (0.53) and 3.49 cm.s^−1^ (1.25), respectively. At least 25 participants will be required in each group (case and control groups) with effect size of 1.04 and 95% power at a 0.05 significance level (G*Power 2014). This number of participants can realistically be recruited from this area if more schools are included in the sampling frame or if schools from additional rural areas are included. The demographics of the participants included in this feasibility study are presented in Table 2. There was no difference between case and control groups for age, height, weight and body mass index for both boys and girls.

**TABLE 2:** The median (range) for age, height, weight and body mass index for the two groups per gender.

Variables	FASD		Control	
	Boys	Girls	Boys	Girls
Age	9 years, 7 months	9 years, 5 months	9 years, 5 months	9 years, 8 months
Height (m)	1.3 (1.1–1.3)	1.3 (1.2–14.3)	1.3 (1.2–1.3)	1.3 (1.2–1.4)
Weight (kg)	24.9 (20.0–27.4)	26.5 (20.0–32.1)	26.3 (24.4–30.0)	28.4 (23.2–47.0)
BMI	16.2 (13.7–18.2)	16.8 (14.6–20.2)	16.9 (14.7–18.6)	18.4 (14.8–22.7)

FASD, foetal alcohol spectrum disorders.

### Measurement instruments

**FIGURE 2:**
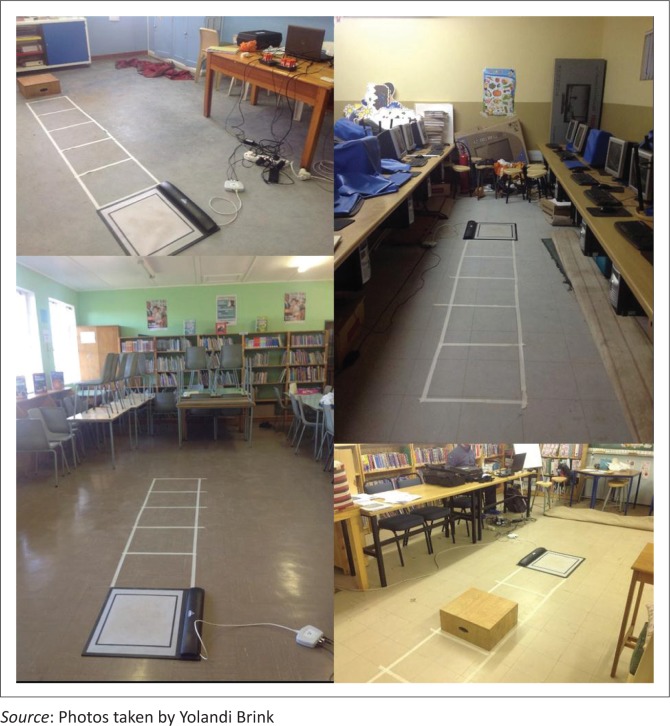
Measurement instrument set-up in different class rooms.

The IMMS and pressure mapping device were portable, and we had no difficulty transporting it to the selected rural schools. Both the IMMS and the pressure mat were easily set-up in spacious and confined classrooms such as computer laboratories. Figure 2 shows some of the different classroom set-ups for this feasibility study.

The set-up and calibration procedure of both instruments were quick to perform within 20 min. The placement of inertial sensors on the participants was easily accomplished and the participants were not hampered by the IMMS or pressure mat while performing the OLS tasks as the inertial sensors are unobtrusive because they are wireless and relatively compact in relation to small children. These two instruments are thus user-friendly to both participant and operator, more time-efficient and cost-effective compared with other similar 3D biomechanical measurement instruments.

### Balance task (one-leg standing)

We were satisfied with the chosen task, that is, OLS with eyes open and closed. However, the visual observation via video recording of the COP displacement revealed that some participants shuffled their feet (change in direction of foot placement on the mat) for one or more of the four conditions. Thus, adjustments will need to be made to the verbal instructions given for the task, that is, ‘place the foot aligned with the pressure mat (and demonstrate) and do not shift the foot from the original placement for the duration of the trial’. A picture of a left and right foot can also be added to the surface of the pressure mat to assist with feet placement in the larger follow-up study.

After reviewing the COP and accelerometry data for OLS with eyes open as provided in Tables 3 and 4, the boys without PAE took longer to achieve postural control (postural sway and mean COP velocity) and at the same time the pelvis segment displayed an increase in activity compared to boys diagnosed with FASD. This difference could possibly be explained when considering gender differences in performing the OLS with eyes open. When viewing the videos of the trials, it was clear that control boys employed a different balance strategy to remain standing on one leg. They either flexed the NWB leg to 90° knee flexion and gradually lowered the foot during the task or only lifted the NWB foot partly of the ground. This balance strategy results in a slightly lower COM, which may have enhanced their ability to maintain balance. It could also be a strategy to improve their ability to make corrections or adapt faster if the line of gravity (LOG) is moved outside of the base of support. However, this caused the NWB hip to abduct to keep the foot off the mat and thus the COM and the LOG shifted more to the NWB side. This probably leads to a more unstable position, increasing strain on the postural stability mechanisms (vestibular, somatosensory and visual) as can be seen by the increase in pelvis segment activity (Table 4). This would likely result in increased postural sway and mean COP velocity to maintain balance. However, this was only true for the boys during eyes-open trials. For the girls, there was no difference in the balance strategy employed while they performed the OLS task with eyes open. Although there was similar pelvis activity displayed between the two groups, the girls without PAE were significantly faster to gain postural control in the anteroposterior (AP) direction.

**TABLE 3a:** The median (range) of the centre of pressure parameters for eyes-open trials.

Variables	COP	All children		Boys		Girls	
		FASD (*n* = 27)	Control (*n* = 26)	FASD (*n* = 16)	Control (*n* = 14)	FASD (*n* = 11)	Control (*n* = 12)
Eyes open	ML_max_ [cm]	2.3 (1.4–3.0)	2.4 (1.7–3.1)	2.2 (1.4–3.0)*	2.7 (1.9–3.1)*	2.4 (1.7–2.9)	2.2 (1.7–2.9)
	AP_max_ [cm]	2.5 (1.8–5.8)	2.9 (1.5–4.9)	2.1 (1.8–4.0)*	3.4 (2.7–4.9)*	3.1 (2.0–5.8)*	2.3 (1.5–3.4)*
	ML_vel_ [cm.s^−1^]	2.3 (1.9–4.1)*	3.3 (1.9–6.5)*	2.5 (1.9–4.1)*	3.4 (2.2–5.8)*	2.3 (2.0–3.1)	2.6 (1.9–6.5)
	AP_vel_ [cm.s^−1^]	4.5 (2.8–10.3)*	6.4 (2.7–30.8)*	4.2 (2.8–10.3)*	6.8 (3.1–10.5)*	4.6 (3.5–7.9)*	6.1 (2.7–30.8)*

FASD, foetal alcohol spectrum disorders.

* , significant difference.

**TABLE 3b:** The median (range) of the centre of pressure parameters for eyes-closed trials.

Variables	COP	All children		Boys		Girls	
		FASD (*n* = 27)	Control (*n* = 19)	FASD (*n* = 16)	Control (*n* = 8)	FASD (*n* = 11)	Control (*n* = 11)
Eyes closed	ML_max_ [cm]	2.7 (1.5–4.5)	2.6 (2.1–4.3)	2.7 (1.9–4.5)	2.8 (2.1–3.7)	2.9 (1.5–4.5)	2.6 (2.1–4.3)
	AP_max_ [cm]	3.7 (1.8–9.2)	3.3 (1.9–7.0)	3.8 (2.4–7.0)	3.4 (1.9–5.5)	3.2 (1.8–9.2)	3.2 (1.9–7.0)
	ML_vel_ [cm.s^−1^]	4.5 (3.1–7.6)*	5.6 (3.0–7.7)*	4.6 (4.1–5.9)	5.7 (3.0–7.7)	4.1 (3.1–7.6)	5.6 (3.3–7.3)
	AP_vel_ [cm.s^−1^]	6.8 (3.9–10.6)	7.6 (4.1–20.5)	6.7 (3.9–9.6)	8.9 (4.1–12.0)	7.1 (5.5–10.6)	7.2 (6.2–20.5)

FASD, foetal alcohol spectrum disorders.

* , significant difference.

**TABLE 4a:** The median (range) of the accelerometry (ACC) parameters for eyes-open trials.

Variables	ACC	All children		Boys		Girls	
		FASD (*n* = 27)	Control (*n* = 26)	FASD (*n* = 16)	Control (*n* = 14)	FASD (*n* = 11)	Control (*n* = 12)
Eyes open	Head ML_ACC_ [mg]	14.2 (7.8–60.4)	16.4 (7.2–37.1)	14.0 (7.8–20.5)*	19.1 (12.1–37.1)*	14.3 (9.7–60.4)	10.1 (7.2–26.0)
	Head AP_ACC_ [mg]	13.7 (8.2–58.0)	15.0 (8.4–52.1)	14.1 (8.9–21.8)*	19.7 (13.5–52.1)*	13.7 (8.2–58.0)	12.2 (8.4–15.6)
	Thorax ML_ACC_ [mg]	12.8 (7.3–26.7)	13.8 (8.6–58.7)	12.4 (7.3–26.7)	17.5 (9.6–58.7)	12.8 (9.1–25.3)	11.7 (8.6–17.7)
	Thorax AP_ACC_ [mg]	12.2 (7.1–25.4)	13.8 (6.4–47.7)	12.3 (7.1–19.8)*	14.9 (12.4–47.7)*	11.8 (7.7–25.4)	11.3 (6.4–14.8)
	Pelvis ML_ACC_ [mg]	14.3 (8.3–31.5)*	19.0 (11.9–65.5)*	14.4 (9.2–30.8)*	27.1 (16.6–65.5)*	13.8 (8.3–31.5)	16.4 (11.9–21.3)
	Pelvis AP_ACC_ [mg]	14.8 (9.0–28.2)*	18.7 (11.0–59.6)*	14.7 (9.2–23.4)*	21.5 (12.1–59.6)*	14.8 (9.0–28.3)	15.4 (11.0–23.2)

**TABLE 4b:** The median (range) of the accelerometry (ACC) parameters for eyes-closed trials.

Variables	ACC	All children		Boys		Girls	
		FASD (*n* = 27)	Control (*n* = 19)	FASD (*n* = 16)	Control (*n* = 8)	FASD (*n* = 11)	Control (*n* = 11)
Eyes closed	Head ML_ACC_ [mg]	25.5 (9.0–113.4)	21.7 (12.6–105.8)	26.2 (13.2–113.4)	29.5 (20.5–105.8)	23.1 (9.0–91.4)	18.6 (12.6–47.0)
	Head AP_ACC_ [mg]	23.8 (10.9–120.1)	21.6 (15.4–146.0)	23.6 (14.6–100.3)	25.8 (16.4–146.0)	25.4 (10.9–120.1)	18.8 (15.4–43.5)
	Thorax ML_ACC_ [mg]	22.7 (10.8–63.0)	19.7 (9.7–31.9)	24.0 (10.9–61.3)	19.7 (13.9–91.9)	21.8 (10.8–63.0)	19.7 (9.7–45.7)
	Thorax AP_ACC_ [mg]	22.0 (10.3–53.8)	19.6 (10.8–77.7)	22.0 (13.5–53.8)	21.6 (16.5–77.7)	24.9 (10.3–32.7)	18.5 (10.8–55.6)
	Pelvis ML_ACC_ [mg]	25.0 (10.7–75.3)	30.7 (15.0–143.2)	26.2 (14.6–75.3)	36.4 (17.8–143.2)	24.1 (10.7–54.1)	26.1 (15.0–86.6)
	Pelvis AP_ACC_ [mg]	24.7 (12.2–71.6)	27.8 (14.4–125.8)	24.0 (13.3–71.6)	27.1 (14.4–125.8)	26.4 (12.2–63.9)	27.8 (15.9–81.0)

FASD, foetal alcohol spectrum disorders.

* , significant difference.

Thus, for the verbal instructions and the visual demonstration by the researcher, more emphasis should be placed on the positioning of the NWB leg, that is, the knee should remain in 90° flexion and if the participant lowers the foot then the trial is unsuccessful and should be recaptured.

The protocol could be further adjusted to include a practice attempt for each task prior to the formal trials. More than two formal attempts could also be allowed (e.g. three to four attempts) if the inclusion criteria for a successful trail is not adhered to; however, there should be a cut-off point for the number of formal attempts as we also consider the capability of the participants to successfully complete all four OLS tasks.

### Preliminary data on one-leg standing analysis

The preliminary data revealed a significant difference between the two groups (children with FASD and controls) for the mean COP velocity and the dynamic acceleration of the pelvis segment during OLS with eyes-open only. These differences were maintained when the groups were subdivided according to gender.

#### Duration of one-leg standing

No trials were excluded when the time variable for both boys and girls for all four conditions were analysed. There was no difference between the left and right sides; thus, the two sides were combined for the eyes-open and -closed trials. The median and ranges for the time variable are shown in Table 5. There was no significant difference between the two groups for the eyes-open trials. However, for the eyes-closed trials, there was a significant difference between case and control groups, by gender, indicating that girls in the control group could stand on one leg significantly longer than affected girls (*p* = 0.041). The relatively better balance control shown by control girls during the eyes-closed trials suggests that they were less dependent on visual input to maintain proper balance compared to case girls (Geuze 2003). However, the gender group sizes are too small to instil confidence that true gender differences exist.

**TABLE 5:** The median (range) in seconds for one-leg standing for eyes-open and -closed trials.

Variables	All children		Boys		Girls	
	FASD (*n* = 28)	Control (*n* = 28)	FASD (*n* = 16)	Control (*n* = 16)	FASD (*n* = 12)	Control (*n* = 12)
Eyes open	19.9 (0.9–20.0) s	19.9 (11.8–20.0) s	19.9 (15.5–20.0) s	19.9 (11.8–20.0) s	19.9 (0.9–20.0) s	19.9 (19.9–20.0) s
Eyes closed	12.8 (2.3–20.0) s	11.8 (1.50–20.0) s	17.4 (3.3–20.0) s	9.8 (1.5–19.9) s	5.2 (2.3–19.9) s*	19.9 (2.1–20.0) s*

FASD, foetal alcohol spectrum disorders.

* , significant difference.

#### Centre of pressure parameters during one-leg standing

For the case group, one trial for eyes open and eyes closed (girls) were unsuccessful and was excluded (*n* = 2). For the control group, two eyes-open (boys) and nine eyes-closed (8 boys and 1 girl) trials were excluded. There was no difference between the left and right sides; thus, the two sides were combined for the eyes-open and -closed trials. The median and range values for the eyes-open and eyes-closed trials for the four COP parameters are shown in Table 3.

**Eyes-open trials:** There was a significant difference between cases and controls for both COP mean velocity parameters (*p* = 0.001). The COP of the control children moved significantly faster in both directions compared to the children from the case group. There were significant differences on all four COP parameters between the case and control boy groups with *p*-values ranging from 0.001 to 0.016. Both the maximum COP displacement and the COP mean velocity were larger for the boys from the control group compared to the case group. There was a significant difference for the AP_max_ parameter between the case and control girl groups (*p* = 0.008). Thus, the girls from the case group showed significantly larger anterior or posterior displacement than control girls. There was also a significant difference between the AP_vel_ parameter between the case and control girls (*p* = 0.049). The COP for the control girls moved significantly faster in the anterior or posterior direction than for the case girls.

**Eyes-closed trials:** There was only a significant difference between the case and control groups for ML_vel_ (*p* = 0.029). The COP of the control children moved significantly faster in the medial or lateral direction than for the case children.

The mean COP velocity is an index of the time-to-postural-control reflecting the amount of movement of the COP within a specified time frame (Benjuya, Melzer & Kaplanski 2004). Our study findings for children without PAE (controls) compare more favourably to previous research on ML_vel_ and less favourably for AP_vel_ as shown by Zumbrunn et al. (2011) who reported a mean ML_vel_ of 3.4 cm.s^−1^ ± 1.2. Zumbrunn et al. (2011) and De Kegel et al. (2011) reported mean AP_vel_ of 3.5 cm.s^−1^ ± 1.3 and 4.9 cm.s^−1^ ± 4.5, respectively, which were lower than the median value reported in our study (AP_vel_ = 6.4 cm.s^−1^; 2.7–30.8) for OLS with eyes open. The mean and standard deviations for this study are reported in Appendix 1 (data not shown). Thus, our children with no PAE (controls) have larger AP_vel_ compared to typically developed children from other countries displaying less efficient postural stability in the AP direction. The mean COP velocity for the control children was also consistently larger than that for the children with FASD in our study while the amount of maximum COP excursion remained similar between the two groups.

Our data for children without PAE compare well with norms data for postural sway (maximum COP excursion) reported by Zumbrunn et al. (2011) for typically developed children aged 8–12 during OLS with eyes open (AP_max_ = 2.3 cm ± 0.7; ML_max_ = 2.2 cm ± 0.6) and Giagazoglou et al. (2013) also tested the postural sway of healthy children (mean age 10.6 ± 1.6) during OLS with eyes open and found AP_max_ = 3.2 cm ± 1.0 and ML_max_ = 1.6 cm ± 0.6. There is consistently greater postural sway in the AP direction compared to ML direction. The larger COP excursion in the AP direction for the girls with FASD indicates less efficient postural control exhibited by the girls with FASD during eyes-open trials.

The increase in COP_max_ and COP_vel_ from an eyes-open to an eyes-closed task is consistent with previous research (De Kegel et al. 2011). Only the study by De Kegel et al. (2011) assessed typically developed children (mean age 9.6 ± 2.0) during OLS with eyes closed and reported a mean AP_vel_ of 9.9 cm.s^−1^ ± 2.4. Our study also found increased AP_vel_ in the eyes-closed condition (median 7.6 cm.s^−1^; 4.1–20.5) than in the eyes-open condition. During the eyes-closed trials, both groups (children with FASD and controls) implemented the same balance strategy (i.e. lifting the NWB leg off the ground in the same manner), which could be the reason why we did not find any difference in the COP or accelerometry outcome measures between the two groups. De Kegel et al. (2011) assessed the reliability of various COP parameters during OLS with eyes-open and -closed and found that eyes-closed trials were less reliable and should not be included in the assessment of postural control of children as a result of large within-person variability during task performance.

#### Accelerometry parameters during one-leg standing

The trials excluded for the COP analysis were also excluded for the analysis of the accelerometry data. The left and right sides were combined for the eyes-open and -closed trials as there was no difference between sides. The median and range values for the eyes-open and eyes-closed trials for the six accelerometry parameters are shown in Table 4.

**Eyes-open trials:** There was a significant difference between the case and control groups for both pelvic accelerometry parameters. The pelvis segment of the control children moved significantly faster in the medial or lateral (*p* = 0.010) and the anterior or posterior (*p* = 0.027) directions than the children from the case group. There were significant differences in accelerometry parameters for five segments (all except Thorax ML_ACC_) between the case and control boy groups with *p*-values ranging from 0.001 to 0.007. The head, thorax and pelvis segments moved faster for the boys from the control group compared to the case group.

**Eyes-closed trials:** There was no significant difference between the case and control groups in any of the accelerometry parameters during the eyes-closed trials.

The 3D accelerometry parameters of the head, thorax and pelvis closely followed the pattern of the mean COP velocity in both directions.

### Limitations

The feasibility sample did not allow for subgrouping other than by gender. We excluded 25% and 42% of eyes-open and eyes-closed trials, respectively, which did not meet the criteria for a successful trial. This is similar to the study done by Johnson et al. (2014) who had 25% of participants not being able to stand on one leg for 3 s but their research protocol allowed participants more attempts until a successful trial was accomplished. In our study we only allowed two attempts as this is a standard protocol to measure children’s ability to perform OLS. We also allowed the children to perform OLS with limited criteria (as explained under the balance task in the methods) in order to observe their balance strategies but this inadvertently compromised the use of all captured trials because it increased the number of unsuccessful trials being excluded.

## Conclusions

The study demonstrated the feasibility of (1) recruiting children with and without FASD in a rural, small town; (2) using the measurement instruments in a real-life, school-based setting and (3) performing the OLS task. There was a difference in mean COP velocity (control group took longer to achieve postural control) and dynamic acceleration of the pelvis (control group displayed an increase in activity in the pelvis segment) between children with FASD compared to children without PAE during OLS with eyes open. This could be attributed to gender differences and the manner in which the OLS task was performed as the difference was mostly found in male participants, with the task being performed differently between male participants in the case and control groups. The primary challenge for the proposed larger study is revision of how the task is performed to ensure that a larger proportion of trials are eligible for inclusion in the data analysis.

### Availability of data and materials

All data sets on which the conclusions of the manuscript rely have been presented in the manuscript and in the additional supporting files.

## APPENDIX 1: The mean (SD) of the COP parameters for eyes open and closed trials.

**TABLE 1a-A1:** The mean (SD) of the centre of pressure parameters for eyes-open trials.

Variables	COP	All children		Boys		Girls	
		FASD *n* = 27	Control *n* = 26	FASD *n* = 16	Control *n* = 14	FASD *n* = 11	Control *n* = 12
Eyes open	ML_max_ [cm]	2.31 (0.41)	2.44 (0.43)	2.24 (0.43)	2.66 (0.39)	2.40 (0.38)	2.11 (0.25)
	AP_max_ [cm]	2.82 (1.03)	2.97 (0.86)	2.45 (0.71)	3.53 (0.63)	3.36 (1.22)	2.35 (0.60)
	ML_vel_ [cm.s^−1^]	2.49 (0.53)	3.49 (1.25)	2.57 (0.61)	3.70 (1.03)	2.39 (0.37)	3.33 (1.54)
	AP_vel_ [cm.s^−1^]	4.72 (1.79)	8.58 (6.39)	4.71 (2.14)	6.80 (2.31)	4.75 (1.20)	11.34 (8.91)

**TABLE 1b-A1:** The mean (SD) of the centre of pressure parameters for eyes-closed trials.

Variables	COP	All children		Boys		Girls	
		FASD *n* = 27	Control *n* = 19	FASD *n* = 16	Control *n* = 8	FASD *n* = 11	Control *n* = 11
Eyes closed	ML_max_ [cm]	2.75 (0.86)	2.84 (0.61)	2.73 (0.70)	2.87 (0.46)	2.78 (1.10)	2.82 (0.72)
	AP_max_ [cm]	3.85 (1.72)	3.53 (1.24)	3.78 (1.31)	3.69 (1.19)	3.94 (2.26)	3.43 (1.32)
	ML_vel_ [cm.s^−1^]	4.54 (1.09)	5.38 (1.34)	4.58 (0.92)	5.52 (1.56)	4.49 (1.35)	5.28 (1.22)
	AP_vel_ [cm.s^−1^]	6.86 (1.79)	9.23 (4.78)	6.48 (1.79)	8.29 (3.23)	7.42 (1.73)	9.91 (5.70)

FASD, foetal alcohol spectrum disorders; COP, centre of pressure.
